# Real-world implications of aphantasia: episodic recall of eyewitnesses with aphantasia is less complete but no less accurate than typical imagers

**DOI:** 10.1098/rsos.231007

**Published:** 2023-10-25

**Authors:** Coral J. Dando, Zacharia Nahouli, Alison Hart, Zoe Pounder

**Affiliations:** ^1^ Department of Psychology, School of Social Science, University of Westminster, London W1B 2HW, UK; ^2^ School of Psychology, University of Derby, Derby, UK; ^3^ Department of Experimental Psychology, University of Oxford, Oxford, UK

**Keywords:** aphantasia, episodic recall, eyewitness memory, reinstatement of context, investigative interviews

## Abstract

Individuals with aphantasia report an inability to voluntarily visually image and reduced episodic memory, yet episodic accounts provided by witnesses and victims are fundamental for criminal justice. Using the mock-witness paradigm, we investigated eyewitness memory of individuals with aphantasia versus typical imagers. Participants viewed a mock crime and 48 hours later were interviewed about the event, randomly allocated to one of three conditions. Two interview conditions included techniques designed to support episodic retrieval mode, namely (i) Mental Reinstatement of Context (MRC) and (ii) Sketch Reinstatement of Context (Sketch-RC). A third Control condition did not include retrieval support. Aphantasic mock-eyewitnesses recalled 30% less correct information and accounts were less complete, but they made no more errors and were as accurate as typical imagers. Interaction effects revealed reduced correct recall and less complete accounts for aphantasic participants in MRC interviews versus Sketch-RC and Control. Aphantaisic participants in the Control outperformed those in both the Sketch-RC and MRC, although Sketch-RC improved completeness by 15% versus MRC. Our pattern of results indicates reduced mental imagery ability might be compensated for by alternative self-initiated cognitive strategies. Findings offer novel insights into episodic recall performance in information gathering interviews when ability to voluntarily visualize is impoverished.

## 1. Introduction

The term ‘episodic memory’ concerns personally experienced events or episodes, which necessarily include contextual information specific to the individual, the time, and place of acquisition [1,2]. Retrieving episodic information is a reconstructive process involving the establishment of episodic retrieval mode. Several cognitive processes are associated with invoking episodic retrieval mode, which is a subjective sense of time, of being the person who experienced the episode, and autonoetic consciousness, a form of consciousness accompanying the act of remembering. Rememberers must relive or re-experience the episode in question by consciously searching for the relevant ‘*what, where* and *when’* information to reconstruct experiences, a process typically likened to mental time travel [1,3,4].

Autonoetic consciousness, or the ‘recollective experience’, involves imaging whereby individuals mentally recreate what has occurred, forming a ‘memory image’ [4,5] whereby more vivid visual imagery correlates with more vivid episodic recall [6–8]. It seems sensible to suggest, therefore, that individuals with reduced mental imagery ability might struggle to invoke episodic retrieval mode. Consequently, the recollective experience may lack ‘richness’ and so recall may be impoverished and comprise more gap-filling errors compared with those with typical imagery ability [9,10]. In some real-world contexts, such as in criminal justice and legal situations, impoverished and erroneous episodic recall can have significant ramifications. Worldwide, criminal justice systems (CJS) rely heavily on episodic accounts from witnesses, victims and survivors^1^ and so erroneous and impoverished accounts necessarily reduce access to justice for witnesses and can undermine criminal investigations, which can result in wrongful convictions [11–13].

Eyewitness memory is of significant national and international interest to applied researchers, legal professionals and government organizations, alike. Yet, a review of the applied experimental memory literature reveals, as far as we can ascertain, very little research on recall performance in information-gathering forensic interviews with individuals who report a reduced ability to visually image. Hence, the real-world implications of being unable or less able to visually image are unclear. Some previous research has used a mock-witness paradigm to investigate recognition performance in a post-event line-up [14] and susceptibility to misinformation [15] when answering a set of leading, forced choice and closed questions. Riske and colleagues [14] found higher imagery vividness was associated with correct recognition in line-ups and Tomes and Katz [15] report that individuals with high vivid imagery were among those more suspectable to misinformation.

Both studies suggest a link between face recognition and directed recall memory and mental imagery, but neither concerned recall memory in an information-gathering interview as advocated in the UK and elsewhere. Accordingly, neither shed light on whether current investigative practice supports or hinders aphantasic witnesses to invoke episodic retrieval mode in interview contexts. Here, we experimentally mirror the experiences of witnesses who report reduced mental imagery. In doing so we investigate the efficacy of the prevalent psychologically guided investigative interview techniques used by professionals to trigger contextual cues encoded alongside the target incident, which are known to scaffold conscious remembering of self-related events [13,16–19].

### 1.1. Aphantasia and cognition

Most people describe their visual imagery experience as a vivid, perception-like experience. Conversely, some otherwise healthy individuals report a lack of ability to visually image, a condition now referred to as aphantasia [20]. Aphantasia is often described as a life without mental images or lacking a ‘mind's eye’ [21] and is believed to be experienced by between 2% and 4% of the population [9,22]. Individuals with aphantasia self-report their ability to mentally image or mentally visualize (these terms are used interchangeably) ranging from being completely absent to being vague or dark whereby aphantasia appears to represent the absence of the ability to voluntarily mentally image.

Visual imagery or ‘*seeing with the mind's eye*’ is widely acknowledged as essential for a range of core cognitive processes [23–25] and so aphantasia can impact future event prospection [6,25] and episodic and autobiographical memory [1,7,10,26,27]. It is unsurprising, therefore, that those who report a lack of ability to visually image can exhibit reduced performance on a range of different cognitive tasks that are heavily reliant upon visual imagery, such as episodic memory, compared with those with typical imagery [27–29]. Self-report studies have revealed people with aphantasia often experience reduced episodic memory, including reduced vividness or richness of episodic re-experiencing [23], poor autobiographical memory, although not always [30,31], and difficulties with face recognition [30,31] compared with those with typical imagery. In addition to self-report data, significant differences have been found using physiological measures which have demonstrated a lack of physiological response when imaging frightening stories and images, believed to be underpinned by an inability to visualize [32,33].

Mental visual imagery is widely accepted as inherent to episodic re-experiencing [7,8,10,34] because it underpins the conscious search for relevant ‘*what, where* and *when’* information [1,3,4]. The importance of contextual information encoded alongside the target episode for cueing conscious remembering of self-related events is widely accepted [17–20]. Context reflects any personally salient elements of an episode, such as the spatial, temporal, environmental or cognitive details, that when available at retrieval improves episodic memory considerably. Therefore, an inability or reduced ability to visually generate contextual details may contribute to impoverished episodic memory via a reduction in contextual cueing. Current practice advocates witnesses be supported to generate contextual details to cue recall at retrieval using empirically validated external recall support techniques, namely Mental Reinstatement of Context [35–37] and Sketch Reinstatement of Context [38–44]. Questions naturally emerge, therefore, centred on both the utility and efficacy of these techniques for aphantasic individuals.

### 1.2. Gathering witness information

Worldwide, witnesses typically provide information during a face-to-face interview, usually conducted by a police officer or investigative professional. In the UK, and elsewhere, the cognitive interview (CI) [45] is one of the prevalent empirically informed interview techniques for eliciting information from witnesses. The CI is a phased recall interview, comprising several retrieval support strategies one being the Mental Reinstatement of Context (MRC) technique. MRC is guided by ‘encoding-specificity’ [46] which posits that reinstating the psychological and physical encoding context at the point of retrieval improves episodic memory.

There is substantial evidence that physically re-experiencing the original encoding context at retrieval can trigger a reliving or re-experiencing of the original event [19,47,48]. It is usually impossible and inappropriate for witnesses to return to the scene of a crime. However, internal cognitive and psychological contextual experiences at encoding can strengthen the encoding–retrieval overlap and have been found to significantly improve memory [47–50]. Accordingly, MRC encourages witnesses to mentally image both the physical and personal context that existed at the time of encoding to facilitate the encoding–retrieval overlap.

MRC comprises a series of verbal instructions, given individually and incrementally at the start of an interview. Witnesses are asked to close their eyes and listen carefully to the instructions, which are all centred on visually imaging various physical experiences and mental states assumed present at encoding. Only then are witnesses asked to verbalize information about the event in question. Therefore, the efficacy of the MRC technique relies heavily upon the ability to construct and maintain numerous mental images over time. The benefits of MRC are clear, whereby MRC improves episodic recall in laboratory and field research compared with interviews when MRC is absent [19,39–41,51,52]. However, MRC has been found to be less effective for older adults and neurodiverse children and adults, because cognitive demand can outstrip cognitive resources available [53–55] resulting in impoverished recall and increased errors [38,39,41–44].

Given MRC relies upon the ability to image, it seems sensible to suggest that MRC might be unsuitable for individuals reporting a lack of ability to visually image, since as with children and older adults, individuals with aphantasia are being asked to undertake a series of cognitive processes that may be too challenging [41,42,53,56]. If so, the potentially positive effects of MRC may be absent. Alternatively, the cognitive effort of attempting to visualize when visual imagery ability is lacking may deplete cognitive resources from self-directed efforts to invoke episodic retrieval mode resulting in significantly reduced recall than might otherwise be the case. While there exists a theoretical and applied impetus for questioning the utility of MRC for individuals with aphantasia, currently there is a dearth of relevant empirical literature.

One alternative that does not include explicit instructions to visually image, is the Sketch Reinstatement of Context (Sketch-RC), which asks witnesses to draw ‘whatever reminds you about what happened’. Drawing personally salient elements of an experienced event apparently encourages a more effortful search through memory, whereby the drawing process seems to trigger contextual cues to facilitate the encoding–retrieval overlap. Sketch-RC has emerged as effective for when interview time pressures preclude the use of MRC, and for the wider witness population, more generally [40–44]. Where participants are interviewed using an interview that uses either the MRC, Sketch-RC, or where external retrieval support is completely absent (Control), findings have consistently revealed a Sketch-RC superiority effect. Recall of correct event units of information is improved and this improvement is not accompanied by a concomitant increase in errors, hence memory accuracy and completeness is enhanced. Consequently, Sketch-RC has recently been integrated into best practice guidance in England and Wales as a suitable technique when interviewing vulnerable witness populations [57].

Visual imagery and visual perception are believed different experiences, albeit they can influence each other [58]. Drawing leverages access to visual mental representations of experienced events without the need to consciously visually image. Individuals with aphantasia appear not to be perception impaired [27,59,60]. Hence drawing at retrieval may prove effective for triggering the encoding–retrieval overlap since drawing a visually perceived event may not necessarily require the same level of conscious mental visual imagery. Indeed, recent research [27] has revealed that although aphantasic participants apparently draw significantly fewer objects at retrieval than controls, they make fewer memory errors during recall. These results lend support to the importance of commonly self-reported verbal strategies used at retrieval and suggest the non-directive nature of the Sketch-RC technique may benefit aphantasic witnesses.

Additional benefits of Sketch-RC emerge from the task support hypothesis [61] which argues memory performance can be improved when retrieval support is uncomplicated and task appropriate [62–64]. Cognitive load can be leveraged by the instructions accompanying a task, which can negatively impact goal-directed performance, here recalling an episode. Accordingly, Sketch-RC comprises just a few straightforward instructions and so reduces dual-task cognitive loading for all witnesses. Perhaps more importantly for individuals with aphantasia, although designed to trigger episodic retrieval mode, Sketch-RC does not explicitly request mental imaging, is non-directive in terms of content, thus allowing individuals to engage in self-initiated, strategic, perceptual search strategies, with little interviewer intervention.

Beyond self-report measures, episodic recall performance of individuals with aphantasia has received relatively scant experimental attention, and mock-witness research that mimics the experiences of witnesses coming onto contact with the criminal justice system has yet to fully consider individuals who report a lack of ability to imagine. Consequently, little is known about real-world memory performance or the utility of current retrieval support techniques for this population. Using the mock-witness paradigm, we manipulate how episodic information is elicited during a post-event investigative interview that follows current investigative practice. In doing so, we control the retrieval support techniques designed to trigger episodic memory at the start of a formal investigative interview, comparing Sketch-RC, MRC and a No Support Control.

The experimental literature relevant to aphantasia in applied information-gathering interview contexts is sparce. However, given self-report data and current theoretical understanding of episodic memory at retrieval, we developed three hypotheses to guide this research and our analysis approach. First, mock eyewitnesses who self-report a lack of ability to visually image will recall less event information than typical imagers during a post-event interview and their recall will be less complete (H^1^). Second, the MRC technique, which relies on the ability to construct and maintain mental images over time, will not improve memory performance participants who report a lack of ability to visually image versus a control (no support) and Sketch-RC interview (H^2^). Finally, the empirical literature indicates that appropriate external support at retrieval improves episodic performance. Sketch-RC does not include explicit instructions to visually image but does offer the opportunity to externalize elements of the search and reconstruction processes thus supporting episodic retrieval mode. Accordingly, Sketch-RC will be most effective, improving memory versus the control (no-support) retrieval (H^3^).

## 2. Methods and materials

### 2.1. Design

A between-subjects experimental design was employed with two independent variables, (i) group with two levels (aphantasia; typical imagers) and (ii) retrieval with three levels (Mental Reinstatement of Context; Sketch Reinstatement of Context; No Support Control). The dependent variable was recall performance measured (i) globally, which refers to recall as a function of the entire interview from start to end and (ii) a function of the free recall and questioning phases of the interview. The amount of correct, erroneous and confabulated units of information verbalized are measured alongside percentage accuracy and completeness (see §§2.5 and 3.1 below).

### 2.2. Participants

Sample size was based on the theoretical centrality of mental imagery and large effect sizes in the small amount of empirical research comparing aphantasics and the general population [22,30]. For 3 × 2 ANOVAs with a large effect size, with *α* = 0.05. and power = 0.8, the projected sample size was a minimum of 15 participants per group (GPower 3.1). We in fact recruited 20 participants from the general population for each group. Hence, a total of 120 adults took part in the research, 47 men and 73 women with a mean age of 33.61 years (s.d. = 8.36 years) ranging from 18 to 51 years. Aphantasic participants were recruited via aphantasia-specific online forums, including ‘Aphantasia (Non-Imager/Mental Blindness) Awareness Group’, ‘Aphantasia!’ and aphantasia discussion pages on Reddit, for example.

Control participants (individuals with typical imagery) were recruited from the general population via online social media sites such as Facebook and Twitter and by word of mouth and snowballing. Aphantasic participants self-identified and further identified by their score on the vividness of visual imagery questionnaire (VVIQ), which measures the vividness of one's visual imagery [19]. Previous studies have differed in the amount of minimal imagery within their classification of aphantasia [22,29]. However, here a wider classification was employed to reflect the applied nature of this research, whereby, in forensic interview contexts, vividness of one's imagery is not considered prior to interview, thus individuals will score across the imagery spectrum. Aphantasic participants with VVIQ scores less than or equal to 32 comprised 18 males and 42 females, mean age: 35.20 years (s.d. = 8.00 years). On the VVIQ, aphantasic participants scored a mean of 17.35 (s.d. = 2.41) ranging from 16 to 28. On the VVIQ, control participants scored a mean of 62.87 (s.d. = 7.92) ranging from 49 to 72. Control participants comprised 29 males and 31 females with mean age 32.02 years (s.d. = 8.48 years). There were non-significant main effects for differences in age across the three retrieval conditions (Control; Sketch-RC; MRC), *F*_2, 114_ = 1.047, *p* = 0.35, or between groups (aphantasic; typical imagers, *F*_1, 114_ = 4.421, *p* = 0.038. The group X condition interaction was also non-significant, *F*_2, 114_ = 0.276, *p* = 0.759.

### 2.3. Procedure and materials

The research project was widely advertised via social media. Potential participants were first contacted via email and provided with information about the research and what would be required of them. A copy of the consent form was provided. Participants were encouraged to contact the research team with any questions prior to deciding whether to participate. Once participants agreed to take part and had asked any questions and consented via email, a suitable time and date for the interview to take place was arranged and participants were allocated an individual participation number, which was used to label their data.

The VVIQ and demographic questions were hosted remotely using Qualtrics. Participants were emailed a one-time link to access the VVIQ, demographic questionnaire and the stimulus video with instructions to watch the video using a laptop or desktop computer. Forty-eight hours later, participants were randomly allocated to one of the three Retrieval conditions, either Mental Reinstatement of Context, Sketch-RC, or No Support Control, and interviewed accordingly (see below). Interviews were conducted face-to-face via a GDPR (2018) compliant video conferencing provider (e.g. Skype, Teams, Google Meets). A 48 h delay between encoding and recall was employed to mirror widespread investigative practice for gathering witness information in cases of non-violent volume crime as depicted in the stimulus event used for this research. Interviews were digitally audio recorded for transcription and coding.

#### 2.3.1. Crime stimulus video

A pre-recorded video lasting 1 min was viewed by participants, individually. The film opens showing a road with numerous cars passing by, and a parade of shops. Two people are seen walking from around a corner, down the road and into one of the shops. Approximately 20 s later, the same two people are seen running out of the shop, and around the corner, chased by a man who is shouting at them. The video then ends.

#### 2.3.2. Interview protocol

All interviews conducted for this research were based on current guidance for eliciting witness information in forensic contexts. As such, each interview comprised two recall phases embedded. First a free recall, which was followed by a probing questioning phase. Questions were asked during this second phase only about topics recalled in the initial free recall phase. All interviews were similarly structured, comprising the following phases: (i) greet, explain, & rapport, (ii) free recall (recall 1), (iii) questioning (recall 2), and (iv) closure. They comprised the same number of retrieval attempts in the same order but differed at the commencement of the free recall phase (only), according to condition, since it was at the start of this first recall phase that the experimental manipulation took place. Five experienced interviewers conducted all interviews, with each interviewer conducting between 14 and 36 interviews each across all conditions, following the condition-appropriate protocols (verbatim) as follows (detailed interview protocols are available from the first author).

1. In the *greet, explain* & *rapport* (common to all interviews) phase interviewers greeted the participant, introduced themselves and explained what the interview would entail. Each participant was given an opportunity to ask any questions, and permission was again sought for each interview to be digitally recorded. Throughout, the interviewer interacted with the participant, contributing as an interested party, using open-ended invitations and associated verbal and physical behaviours to exchange information and to demonstrate an understanding of the situation from the participant's point of view [65–67].
2. The *free recall* phase, referred to as recall phase one, is common to all interviews, invites participants to ‘explain’ everything they can recall about the experienced event using an open-ended invitation. This differed across conditions as follows. In the Sketch Reinstatement of Context condition (Sketch-RC) participants were first asked to draw the to-be-remembered event in as much detail as possible [39,40]. Participants were instructed to draw anything that reminded them of what happened. Participants were given unlimited time to draw. Once participants had finished, the interviewer then verbalized four retrieval instructions: (i) I only want you to tell me what you *actually* remember, please don't guess, (ii) if you can't remember just say so, (iii) tell me absolutely everything you can, even if you can only remember partial details, or apparently insignificant information, and (iv) tell me if you do not understand what I am asking or to repeat the question. From here on these four instructions are referred to as the Retrieval Instructions.
  In the Mental Reinstatement of Context (MRC) condition the interviewer gave instructions aimed at aiding the participant to mentally reinstate both the physical and psychological context that existed at the time of encoding in line with the procedure currently taught to police interviewers. The instructions were delivered slowly and deliberately, and in between each instruction the interviewer paused for 5 s to allow enough time for the participant to picture/image, and reinstate the context as instructed. Participants were then given the Retrieval Instructions.
  In the No Support condition, the interviewer provided no retrieval support, but simply verbalized the Retrieval Instructions. Irrespective of condition, participants were given unlimited time to explain what they remembered, during which time they were uninterrupted by the interviewer. Throughout, the interviewer displayed supportive and active listening behaviour, while making brief bullet notes about the main topics remembered, and the order of those topics as they were verbalized by the interviewee (for use in the questioning phase).
3. The *questioning* phase, (recall phase two—common to all interviews) immediately followed. All participants were again given the Retrieval Instructions prior to the commencement of this phase, during which the interviewer questioned each participant using a tell, explain, or describe question, *only* about each of the topics recalled and in the order in which they were recalled during the initial free recall phase. To do this, the interviewer used the notes made during that free recall phase.
4. Thereafter, the interviewer completed the *closure* phase, during which the participant was thanked for his/her participation, debriefed and offered an opportunity to ask questions.

### 2.4. Memory coding

Interviews were digitally audio and video recorded and transcribed verbatim. Transcriptions were first coded for event Units of Information (UoI) by two independent coders. For example, five individual UoI are underlined in the following verbal recall, ‘*the man had short dark hair and was wearing blue jeans*'. A total of 71 event UoI were identified with reference to the stimulus event, resulting in a coding template comprising 71 unique correct UoI. Using the coding template, participants' recall of UoI were coded as either correct (occurred in the event and so is listed on the coding template and was correctly recalled), erroneous (occurred in the stimulus event in part but described with some error, e.g. describing a brown jacket, when in fact the jacket was black), or confabulated UoI (reporting information that was not present in the event). UoI were only scored once. Repetitions were not scored irrespective of interview phase.

Twenty interviews from each condition were randomly selected for coding by two independent coders blind to the aims and hypotheses of the research but familiar with the method of scoring. Two-way mixed effects intraclass correlation coefficient (ICC) analysis testing for absolute agreement between coders for the overall amount of correct, erroneous and confabulated recall UoI was conducted. Mean estimations with 95% CI reveal very good inter-rater reliability for correct information, ICC = 0.899 (95% CI 0.593; 0.975), errors, ICC = 0.979 (95% CI 0.948; 0.992), and confabulations, ICC = 0.865 (95% CI 0.498; 0.964).

### 2.5. Interviewer adherence and manipulation analyses

Two interviews per condition from each of the five interviewers (30 in total) were randomly selected and coded for interviewer adherence to the condition-relevant verbal protocols. Each interview was coded by two independent coders blind to the aims and hypotheses of the research. Coders scored each of the relevant behaviours as absent (scored 0), partially present (scored 1) and fully present (scored 2). Prior to coding, coders participated in a training session held by the first author, during which the interview protocols and coding system were explained. Coders then practiced coding and discussed any disagreements/misunderstandings with the trainer to reach a consensus using the training interviews.

Irrespective of interview condition, all protocols included (i) engage and explain, (ii) retrieval instructions, (iii) free recall, (iv) questioning, and (v) closure. The Sketch Reinstatement of Context and Mental Reinstatement of Context interviews included additional condition specific instructions. Two-way mixed effects intraclass correlation coefficient (ICC) analysis testing for absolute agreement between coders indicated good/very good inter-rater reliability for all interviewer behaviours: engage and explain, ICC = 0.851 (95% CI, -0.415; 0.305), retrieval instructions, ICC = 1.00 (95% CI, -0.388; 0.388), free recall, ICC = 0.920 (95% CI, -0.449; 0.362), questioning, ICC = 0.806 (95% CI, -0.500; 0.300), and closure, ICC = 0.892 (95% CI, -0.454; 0.345), Sketch Reinstatement of Context, ICC = 0.750 (95% CI, -0.944; 0.611), and Mental Reinstatement of Context, ICC = 0.778 (95% CI, -0.865; 0.579).

Kruskal–Wallis *H* tests for the five interview instructions/techniques common to all conditions revealed non-significant differences across conditions for the presence/absence of the individual behaviours, all *H*s (2) < 3.090, all *p*s > 0.213, hence all were similarly present/absent. Interviews in the Sketch Reinstatement of Context and Mental Reinstatement of Context conditions comprised an additional condition-specific retrieval technique. As expected, significant differences emerged across conditions for Sketch Reinstatement of Context, *H* (2) = 28.370, *p* < 0.001, and Mental Reinstatement of Context, *H* (2) = 28.482, *p* < 0.001. *Post hoc* tests revealed the Sketch Reinstatement of Context and Mental Reinstatement of Context were present only in the relevant interview condition, hence each manipulation was applied correctly according to condition, all *p*s < 0.001

## 3. Results

### 3.1. Analysis approach

A series of 2 (Aphantasia; Control) × 3 (Mental Reinstatement of Context; Sketch Reinstatement of Context; No Support) ANOVAs were conducted applying a Bonferroni's corrected alpha as appropriate. Global memory performance main effects and interactions for the number of correct, incorrect and confabulated items recalled, and percentage accuracy were analysed. Percentage accuracy is calculated by summing all the event UoI recalled, including all verbalized correct, errors and confabulations, and then dividing total correct UoI by the sum of error UoI plus confabulation UoI. We further analysed memory performance as a function of the free recall and questioning phases, and percentage completeness of recall in terms of how many of the possible correct UoI were recalled. This analysis approach offers a nuanced understanding of the impact of the experimental manipulations and group differences.

### 3.2. Global memory performance

#### 3.2.1. Global correct recall

There were significant main effects of group, *F*_1, 114_ = 50.81, *p* < 0.001, $\eta_{p}^{2}=0.31$, and condition, *F*_2, 114_ = 15.11, *p* < 0.001, $\eta_{p}^{2}=0.21$. Aphantasics recalled fewer correct UoI than typical imagers (see table 1 for global performance main effects and interaction means, s.d. and 95% CIs). Participants in the Sketch-RC recalled more correct UoI than those in both the MRC, *p* < 0.001 and Control, conditions, *p* = 0.002. Participants in the Control condition recalled more correct UoI that the MRC, *p* = 0.002.

**Table 1.** Global UoI memory performance (*N* = 120) means, s.d.s and 95% CI for main effects and interactions as a function of group (aphantasia; typical imagers) and condition (MRC; Sketch-RC; Control).

	mean (s.d.) 95% CI			
	correct	errors	confabulations	% accuracy
aphantasic	29.78 (7.62) 28.19, 31.38	3.92 (1.75) 3.46, 4.37	.93 (.90) 0.69, 1.18	85.50 (5.96) 83.07, 87.93
typical imagers	37.88 (6.96) 36.29, 39.48	3.48 (1.81) 3.03, 3.94	.90 (.88) 0.66, 1.15	89.64 (12.28) 85.62, 90.48
Sketch-RC	37.82 (6.96) 35.88, 39.78	4.05 (2.10) 3.49, 4.60	1.03 (1.02) 0.73, 1.33	88.07 (5.98) 85.10, 91.05
MRC	30.20 (9.24) 28.25, 32.15	3.83 (1.43) 3.27, 4.38	.88 (.88) 0.73, 1.33	85.70 (5.94) 82.72, 88.68
Control	33.47 ( 6.92) 31.53, 35,43	3.23 (1.70) 2.67, 3.78	.85 (.92) 0.55, 1.15	86.55 (14.59) 83.57, 89.53
aphantasic Sketch-RC	34.65 (6.80) 31.89, 37.41	4.35 (1.72) 3.56, 5.13	1.05 (.99) 0.63, 1.47	86.30 (6.09) 82.09, 90.51
aphantasic MRC	22.85 (5.44) 20.09, 25.61	4.00 (1.37) 3.21, 4.79	.90 (.85) 0.48, 1.32	82.00 (4.96) 77.79, 86.21
aphantasic Control	31.85 (4.95) 29.09, 34.61	3.40 (2.04) 2.61, 4.19	.85 (.98) 0.43, 1.27	88.20 (5.23) 83.99, 92.41
typical imagers Sketch-RC	41.00 (5.66) 38.24, 43.76	3.75 (2.43) 2.96, 4.54	1.00 (1.01) 0.58, 1.42	89.85 (5.44) 85.61, 94.06
typical imagers MRC	37.55 (5.64) 34.79, 40.31	3.65 (1.50) 2.86, 4.44	.85 (.93).43, 1.27	89.40 (4.36) 85.19, 93.61
typical imagers Control	35.10 (8.25) 32.34, 37.86.	3.05 (1.32) 2.26, 3.84	.85 (.92) 0.43, 1.27	84.90 (20.09) 80.69, 89.11

There was a significant group × condition interaction, *F*_2, 114_ = 9.05, *p* < 0.001, $\eta_{p}^{2}=0.14$. Aphantasics in the Sketch-RC condition recalled more correct UoI than those in the MRC, *p* < 0.001, with no significant difference between the Sketch-RC and Control, *p* = 0.158. Aphantasic participants in the Control recalled more correct UoI than those in the MRC, *p* < 0.001, but fewer correct UoI than typical imagers in both the MRC, *p* < 0.001, and Sketch-RC, *p* = 0.002, conditions. No significant difference emerged between the groups in the Control condition, *p* = 0.101 (see table 1).

#### 3.2.2. Global erroneous recall

There were non-significant main effects of group, *F*_1, 114_ = 1.79, *p* = 0.183, and condition, *F*_2, 114_ = 2.313, *p* = 0.104. The group × condition interaction was also non-significant, *F*_2, 114_ = 0.066, *p* = 0.936.

#### 3.2.3. Global confabulations

There were non-significant main effects of group, *F*_1, 114_ = 0.036, *p* = 0.849, and condition, *F*_2, 114_ = 0.391, *p* = 0.677. The group × condition interaction was also non-significant, *F*_2, 114_ = 0.009, *p* = 0.991.

#### 3.2.4. Global percentage accuracy

Main effects of group, *F*_1, 114_ = 2.160, *p* = 0.144, and condition, *F*_2, 114_ = 0.641, *p* = 0.528, were non-significant. The group × condition interaction was also non-significant (applying corrected alpha 0.017), *F*_2, 114_ = 3.352, *p* = 0.042.

### 3.3. Completeness

**Figure 1.**
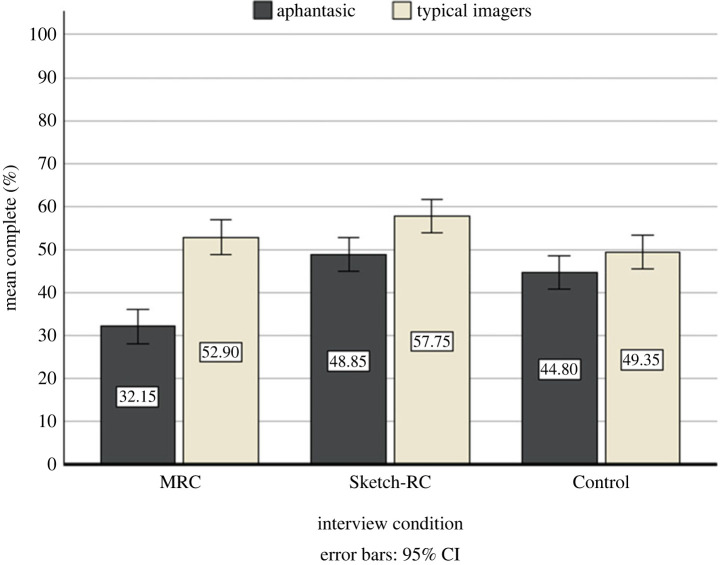
Mean percentage UoI completeness group (aphantasia; typical imagers) and condition (MRC; Sketch-RC; Control) interactions (*N* = 120).

There were significant main effects of group, *F*_1, 114_ = 50.13, *p* < 0.001, $\eta_{p}^{2}=0.31$, and condition, *F*_2, 114_ = 15.05, *p* < 0.001, $\eta_{p}^{2}=0.21$ (see figure 1). Aphantasic participant recall was less complete than typical imagers (*M*_Aphantasia_ = 41.93, s.d. = 10.77, 95% CI 39.68, 44.19; *M*_Typical_ = 53.33, s.d. = 9.89, 95% CI 51.08, 55.59). All participants in the Sketch-RC (*M*_Sketch*-RC*_ = 53.30, s.d. = 9.85, 95% CI 50.54, 56.06) and Control (*M*_Control_ = 47.07, SD = 9.81, 95% CI 44.31, 49.84) were more complete than those in the MRC, *p* < 0.023 (*M*_MRC_ = 42.52, s.d. = 13.04, 95% CI 39.76, 45.29). Participants in the Sketch-RC were more complete than the Control, *p* = 0.002.

The group × condition interaction was also significant, *F*_2, 114_ = 9.039, *p* < 0.001, $\eta_{p}^{2}=0.14$ (see figure 1). Aphantasics in the Sketch-RC and Control were more complete than aphantasics in the MRC condition, *p* < 0.001, with no difference detected between the latter two conditions, *p* = 0.140. Aphantasics were less complete than typical imagers in both the Sketch-RC and MRC conditions, all *p*s < 0.002. No difference was detected between aphantasics and typical imagers for completeness in the Control condition, *p* = 0.106. Typical imagers in the Sketch-RC were more complete than typical imagers in the Control, *p* = 0.003. No difference was detected for completeness between the Sketch-RC and MRC, *p* = 0.085 or the MRC and Control, *p* = 0.206.

### 3.4. Free recall phase

#### 3.4.1. Correct

There were significant main effects of group, *F*_1, 114_ = 42.64, *p* < 0.001, $\eta_{p}^{2}=0.27$, and condition, *F*_2, 114_ = 21.77, *p* < 0.001, $\eta_{p}^{2}=0.28$, and a significant group × condition interaction, *F*_2, 114_ = 10.42, *p* < 0.001, $\eta_{p}^{2}=0.15$ for correct UoI in the first (free recall) phase of interviews. Aphantasics recalled fewer correct UoI (*M* = 17.27, s.d. = 6.22, 95% CI 15.70, 18.83) than typical imagers (*M* = 24.55, s.d. = 8.61, 95% CI, 22.98, 26.11). Participants in the Sketch-RC condition (*M* = 25.80, s.d. = 9.29, 95% CI 23.89, 27.71) recalled significantly more correct UoI than those in the MRC (*M* = 16.92, s.d. = 7.71, 95% CI 15.01, 18.84) and Control (*M* = 20.00, s.d. = 4.89, 95% CI 18.07, 21.91), *p* < 0.001. All participants in the Control condition recalled more correct UoI than those in the MRC, *p* = 0.027.

Non-significant differences emerged between aphantasics and those with typical imagery in the Control condition, *p* = 0.959 (see table 2 for interaction means, s.d.s and 95% CIs). However, aphantasics in both the Sketch-RC and MRC conditions recalled fewer correct UoI than participants with typical imagery, all *p*s < 0.001. Aphantasics in the MRC condition recalled less UoI than those in both the Control and Sketch-RC conditions, all *p*s < 0.001, with a non-significant difference between Sketch-RC and Control, *p* = 0.918.

**Table 2.** Free recall and questioning phase recall for group × condition UoI interactions (*N* = 120) means, s.d.s and 95% C.I.

	mean (s.d.) 95% CI		
	correct	errors	confabulations
MRC free recall			
aphantasic	11.70 (4.71) 8.87, 14.43	1.50 (.61) 1.05, 1.95	.30 (.47) 0.05, 0.55
typical imagers	22.15 (6.52) 19.42, 24.88	1.55 (.83) 1.09, 2.00	.40 (.50) 0.15, 0.65
MRC questioning			
aphantasic	11.15 (6.67) 8.02, 14.29	2.50 (1.19) 1.93, 3.07	.30 (.47) 0.05, 0.55
typical imagers	15.40 (8.13) 12.26, 18.84	2.10 (1.02) 1.53, 2.67	.40 (.50) 0.15, 0.65
Sketch-RC free recall			
aphantasic	20.15 (5.56) 17.42, 22.88	1.65 (1.46) 1.20, 2.10	.55 (.68) 0.30, 0.80
typical imagers	31.95 (9.09) 29.22, 34.68	1.55 (1.28) 1.10, 2.00	.45 (.60) 0.20, 0.70
Sketch-RC questioning			
aphantasic	14.50 (6.25) 11.36, 17.64	2.75 (1.21) 2.18, 3.32	.55 (.69) 0.30, 0.80
typical imagers	9.55 (9.66) 6.41, 12.69	2.2 (1.64) 1.63, 2.77	.45 (.60) 0.20, 70
Control free recall			
aphantasic	19.95 (4.26) 17.22, 22.68	1.25 (.97) 0.80, 1.70	.30 (.47) 0.05, 0.55
typical imagers	20.05 (5.55) 17.23, 22.78	1.25 (.72) 0.80, 1.70	.45 (.61) 0.20, 0.70
Control questioning			
aphantasic	11.90 (5.67) 8.76, 15.04	2.15 (1.50) 1.58, 2.72	.30 (.47) 0.05, 0.55
typical imagers	15.05 (4.98) 11.91, 18.19	1.80 (1.10) 1.23, 2.37	.45 (.60) 0.20, 0.70

#### 3.4.2. Erroneous

There were non-significant main effects of group, *F*_1, 114_ = 0.008, *p* = 0.929, and condition, *F*_2, 114_ = 1.302, *p* = 0.276, for erroneous UoI. The group × condition interaction was also non-significant, *F*_2, 114_ = 0.056, *p* = 0.946.

#### 3.4.3. Confabulated

There were non-significant main effects of group, *F*_1, 114_ = 0.237, *p* = 0.627, and condition, *F*_2, 114_ = 0.817, *p* = 0.444, for confabulated UoI. The group × condition interaction was also non-significant, *F*_2, 114_ = 0.552, *p* = 0.577.

### 3.5. Questioning phase

#### 3.5.1. Correct

There were non-significant main effects of group, *F*_1, 114_ = 0.399, *p* = 0.529, (*M*_aphantasia_ = 12.51, s.d. = 6.30, 95% CI 10.70, 14.33; *M*_typical_ = 13.33, s.d. = 8.16, 95% CI 11.52, 15.14) and condition, *F*_2, 114_ = 0.492, *p* = 0.613, (*M*_Sketch-RC_ = 12.02, s.d. = 8.41, 95% CI 9.81, 14.24; *M*_MRC_ = 13.28, s.d. = 7.69, 95% CI 11.06, 15.49; *M*_Control_ = 13.47, s.d. = 5.50, 95% CI 11.26, 15.69). However, a significant group × condition interaction emerged in this second recall phase, *F*_2, 114_ = 5.03, *p* = 0.008 (see table 2 for interaction means, s.d.s and 95% CIs). Aphantasics in the Sketch-RC recalled more correct UoI than those in the MRC and Control, *p* < 0.029, with non-significant differences between the latter two conditions*, p* = 0.162. Typical imagers in the Sketch-RC also recalled more correct UoI in the questioning phase than those in the MRC and Control, *p*s < 0.016, with a non-significant difference between the latter two conditions, *p* = 0.876.

#### 3.5.2. Erroneous

There were non-significant main effects of group, *F*_1, 114_ = 3.354, *p* = 0.070, (*M*_aphantasia_ = 2.47, s.d. = 1.31, 95% CI 2.13, 2.80; *M*_typical_ = 2.03, s.d. = 1.27, 95% CI 1.70, 2.36) and condition, *F*_2, 114_ = 1.533, *p* = 0.220, for erroneous UoI, (*M*_Sketch-RC_ = 2.47, s.d. = 2.48, 95% CI 2.07, 2.88; *M*_MRC_ = 2.30, s.d. = 2.30, 95% CI 1.89, 2.71; *M*_Control_ = 1.97, s.d. = 1.98, 95% CI 1.57, 2.38). The group × condition interaction was also non-significant, *F*_2, 114_ = 0.064, *p* = 0.938.

#### 3.5.3. Confabulated

There were non-significant main effects of group, *F*_1, 114_ = 0.386, *p* = 0.535 (*M*_aphantasia_ = 0.55, s.d. = 0.67, 95% CI 0.36, 0.74; *M*_typical_ = 0.47, s.d. = 0.77, 95% CI 0.28, 0.65) and condition, *F*_2, 114_ = 0.062, *p* = 0.940, for confabulated UoI, (*M*_Sketch-RC_ = 0.52, s.d. = 0.75, 95% CI 0.29, 0.75; *M*_MRC_ = 0.25, s.d. = 0.75, CI 0.29, 0.75; *M*_Control_ = 0.47, s.d. = 0.68, 95% CI 0.24, 0.70). The group × condition interaction was also non-significant, *F*_2, 114_ = 0.247, *p* = 0.781.

## 4. Discussion

Towards better understanding the real-world implications of aphantasia, we employed a mock-witness paradigm to mirror the experiences of witnesses when recounting what they have seen and heard. In doing so, we manipulated episodic elicitation protocols in line with investigative practice in the UK and elsewhere. Drawing on the prevalent psychologically guided retrieval techniques designed to trigger episodic memory at the start of a formal forensic interview, we considered the efficacy, or otherwise, of the Sketch-RC and MRC, versus a no retrieval support Control. As far as we are aware, this research is novel in that individuals with aphantasia have yet to participate in applied experimental research concerned with recall performance using elements of the cognitive interview. Hence, findings offer insights into the impact of a reduced ability to visually image in terms of the quality and quantity of episodic information recalled and shed light on a need to better understand how to support *all* individuals at the point of retrieval to trigger ‘best’ memorial performance.

We hypothesized that mock eyewitnesses who self-reported a lack of ability to visually image would recall fewer units of event information and that their accounts would be less complete than typical imagers, since autonoetic consciousness, or the ‘recollective experience’, is believed to involve forming a ‘memory image’. Objective content-driven analysis of memorial performance supported this hypothesis. Irrespective of retrieval interview condition, aphantasic participants recalled 30% fewer correct units of episodic information items than typical imagers, and their accounts were over 10% less complete. These main effect findings concur with self-report data concerning reduced ability to remember episodic events [23]. However, given the experimental paradigm employed here, teasing apart whether impoverished recall reflects a failure of relational binding at encoding or an inability to access and associate visual information to cue retrieval is impossible [68], although see [32]. That said, contextual binding theory and temporal context model of episodic memory both predict the most relevant cues at retrieval are those associated with the physical and personal context at the time of encoding [69,70]. Reduced ability to mentally image/recreate the physical and personal context at retrieval may account, in part, for our findings, since activation of cues play a central role in the reconstruction of episodes in an ‘experience-near’ manner.

Of note is that irrespective of retrieval condition, we did not find any differences for erroneous recall (confabulations and errors) as a function of group, although as the power calculation indicates, our sample size was not powerful enough to detect small or medium effects. Nonetheless, theoretically and from an applied perspective, this finding suggests reduced ability to visually image may have impacted post-encoding retrieval processes rather than the encoding processes themselves, on two counts. First, in forensic interview contexts, when encoding is compromised (for whatever reason) individuals typically recognize their event memory is poor when responding to questions. In such instances, impoverished correct recall is often accompanied by increased errors [68,69] triggered by real and/or perceived external demand characteristics, which occur in experimental paradigms as employed here and in the real world. Wanting to perform well in terms of verbalizing a lot of event information can serve to inflate errors when memory is poor [70–72] because individuals have a tendency to ‘gap fill’ using schematic/script guided retrieval and/or guess, for example [73,74].

Second, although retrieval cues are thought to ‘map’ onto event knowledge for specific episodes, it is argued that individuals also consciously draw on memory for previously experienced episodes to assist in reconstructing the to-be-remembered event [26,75,76]. It seems sensible to suspect that aphantasic individuals may have a reduced ‘pool’ of episodes upon which to draw. The literature argues [77,78] visual memory imagery is an essential component of the phenomenology of episodic recollection and so reduced visual imagery seems likely to have a negative impact on episodic memory, which is borne out by self-reports. That said, remembering is a dynamic process influenced by numerous variables that can impact post-encoding retrieval, such that memories can alter over time, and so more research concerned with independently manipulating the encoding and retrieval processes would be beneficial towards unpacking our pattern of results. Nonetheless, from an investigative and criminal justice perspective, erroneous recall of units of information (UoI) is arguably more damaging than reduced quantity of correct UoI. Here, overall percentage accuracy was not found to differ significantly across groups, which is an important distinction. ‘Good’ quality recall is marked by a high volume of correct UoI recall, accompanied by very few error or confabulated UoI, which is the pattern of results found here.

Our second hypothesis was that the MRC external support technique, which relies on the ability to construct and maintain mental images over time, would not improve memory performance for those reporting a lack of ability to visually image. Unsurprisingly, because MRC relies on the ability to construct and maintain mental images over time, our results support this hypothesis. Aphantasic participants recalled almost 40% less correct items of event information in the MRC interview condition than in the No Support Control, again with no difference in the number of errors or confabulations. This pattern of results was consistent when considering performance globally (across the entire interview comprising two recall attempts) and the recall phases alone. External retrieval support in the form of the MRC appears to have disrupted self-initiated cognitive strategies for reconstructing episodic information, possibly diverting cognitive resources away from remembering towards trying to image.

When aphantaisic participants were ‘allowed’ to retrieve and reconstruct the event in the absence of external retrieval support, as in the Control condition, episodic recall significantly improved as did accuracy and completeness. Our results indicate, therefore, that any structured episodic retrieval interview that focuses on imagery may interfere with non-visual compensatory strategies, albeit such strategies are not yet understood. It has been suggested that aphantasics might store information in visual working memory without conscious awareness [26]. Further, that a lack of mental imagery might be compensated for under some conditions by way of alternative cognitive strategies, but reduced metacognitive insight impedes understanding [79]. Indeed, some aphantasic individuals have been reported to be highly imaginative, and able to complete tasks that were previously believed to rely on visual imagery, indicating visualization may not be the only technique for triggering episodic re-experiencing [80].

Finally, we hypothesized that the Sketch-RC would improve performance versus no support at retrieval. Our results did not fully support this hypothesis. The applied literature indicates the importance of appropriate external support for invoking episodic retrieval mode, and so we expected drawing might improve the encoding–retrieval overlap [39,42–44,76]. Drawing a visually perceived event does not necessarily require conscious mental visual imagery, and Sketch-RC does not include explicit instructions to visually image. Yet, this approach was not completely effective for individuals lacking the ability to visually image. We did not find a significant improvement for the amount of correct UoI, likewise completeness and accuracy did not statistically differ from the Control interviews. Why is unclear. Offering opportunities to externalize elements of the search and reconstruction processes has been found to be effective for improving witness memory versus a No Support Control and MRC retrieval condition in the general adult population, for children, older adults and children with autism [38–42]. While we did not control for autistic traits in our aphantasic sample, people with aphantasia have been shown to score higher for autistic traits than typical imagers [30].

The retrieval strategies used by aphantasic individuals are not understood, and so future research should consider ways to better understand the nature of autonoetic consciousness in this population. Of the two external support techniques employed here (MRC and Sketch-RC) neither cued erroneous patterns of activation, although MRC resulted in reduced correct recall, and accounts were far less complete. Sketch-RC on the other hand improved completeness in aphantasic participants by approximately 15% versus the MRC interview and had no detrimental effect on accuracy. This offers further evidence using objective and subjective data streams rather than self-report data that aphantasia is associated with a diminished ability to re-experience the past and simulate the future, and that visual imagery is an important cognitive tool for the dynamic retrieval of episodic details.

Cognitive offloading refers to our reliance on the external environment to reduce cognitive demand at retrieval, and it appears that counter to our expectations, sketching at retrieval was not an effective offloading technique for individuals that report a lack of ability to visually image. Although metacognition was not the focus of the research reported here, our results also indicate metacognitive insight may not be lacking, since poor metamemory monitoring is typically associated with increased recall errors, which did not emerge here [81–83]. However, further research is necessary to tease apart metamemory processes in this population.

Forensic interviews with witnesses comprise several recall phases designed to maximize retrieval performance, simultaneously controlling the retrieval process to reduce contamination of the memory trace. Here, we mirrored good practice. First, participants were asked to provide a freely recalled account of the experienced event. This initial account was followed by probing questions guided by information recalled during the initial free recall, only. Hence, the initial free recall is a fundamental element of a forensic interview since it guides the follow-on probing questions. Analysis of the initial free recall phase revealed aphantasic participants' recall of correct UoI mirrored that of typical imagers in the Control but was significantly less than those in the Sketch-RC and MRC conditions. Visual mental imagery is described as a depictive internal representation, akin to a weak form of perception, but in the absence of external stimuli [10,83,84] which triggers a re-experiencing of a version of past events. Here, sketching was apparently no more effective for triggering internal representations of the target event than self-initiated strategies [85].

Turning to the questioning phase, generally our pattern of results for this phase mirrored the global and free recall phase findings, apart from one notable exception. Aphantasic participants in the Sketch-RC condition recalled more correct UoI in this phase than the other two retrieval conditions. There may have been a Sketch-RC carry-over effect whereby the sketching process had in some way consolidated the memory trace, making it more robust, as has been reported elsewhere. However, more research is needed to replicate and better understand the impact of sketching for consolidation following a 48 h delay. However, in this phase no differences emerged between the Control and MRC offering some indication of the importance of appropriate techniques for triggering the recollective experience at retrieval, versus nothing.

As is the case with all experimental research of this nature, there are several clear limitations, which are not unique, but should be borne in mind when interpreting the findings. Mock-witness paradigms do not precisely replicate the experiences of real eyewitnesses. Nonetheless, some social and cognitive demands were present. Participants were recruited from the general population and were aware that memory was the topic of this research, and that memory would be assessed. This demand characteristic is present with real witnesses, albeit to a greater degree, who understand the importance of their memory performance [86–88] and the need to provide detailed information. Aphantasic participants from the general population are hard to reach. *A priori* power analysis [89] revealed our size was adequate to detect large effects, but not powerful enough to detect small effects and so future research might consider how to reach out to encourage wider participation towards larger sample sizes to allow a more nuanced understanding. However, the impact of small effect sizes for applied research is currently the subject of discussion [90,91].

Despite the limitations, our research offers novel insight into the challenges of episodic remembering for individuals who report a lack of ability to visualize, and again highlights the importance of better understanding diverse witness populations towards developing population appropriate task support for complex cognition in applied forensic settings. Impoverished episodic recall limits access to justice, since memory serves as evidence in criminal and civil legal proceedings. What can be *retrieved* from that memory is determined by a multitude of factors, but appropriate techniques for supporting the encoding retrieval overlap are known to be important for improved performance.

## Data Availability

Raw data file has been deposited in the OSF, available via this link: https://osf.io/dv456/?view_only=bc27c9a1df4846c8a2ffb69ca19b3541.

## Footnotes

1 From here on we use the terms witness and eyewitness to refer to witnesses, victims and survivors of crime.
